# A digital lifestyle behaviour change intervention for the prevention of type 2 diabetes: a qualitative study exploring intuitive engagement with real-time glucose and physical activity feedback

**DOI:** 10.1186/s12889-020-09740-z

**Published:** 2021-01-12

**Authors:** Maxine E. Whelan, Francesca Denton, Claire L. A. Bourne, Andrew P. Kingsnorth, Lauren B. Sherar, Mark W. Orme, Dale W. Esliger

**Affiliations:** 1 Centre for Intelligent Healthcare, Coventry University, Coventry, UK; 2 National Centre for Sport and Exercise Medicine, Loughborough University, Loughborough, UK; 3 School of Sport, Exercise and Health Sciences, Loughborough University, Loughborough, UK; 4 Centre for Exercise and Rehabilitation Science, NIHR Leicester Biomedical Research Centre-Respiratory, Leicester, UK; 5 NIHR Leicester Biomedical Research Centre-Lifestyle, Leicestershire, UK; 6 Department of Respiratory Sciences, University of Leicester, Leicester, UK

**Keywords:** Qualitative, Self-monitoring, Glucose, Physical activity, Wearables, Diabetes prevention

## Abstract

**Background:**

Mobile health technologies have advanced to now allow monitoring of the acute physiological responses to lifestyle behaviours. Our aim was to explore how people engaged with real-time feedback on their physical activity and glucose levels over several weeks.

**Methods:**

Semi-structured interviews with 26 participants (61.5% female, 56.6 years) at moderate-to-high risk of developing type 2 diabetes were conducted. Interviews were completed after participants took part in an intervention comprising a flash glucose monitor (Freestyle Libre) and a physical activity monitor (Fitbit Charge 2). Purposive sampling ensured representation of ages, genders and group allocations.

**Results:**

Inductive thematic analysis revealed how individuals intuitively used, interpreted and acted on feedback from wearable technologies. Six key themes emerged: triggers of engagement with the technologies, links between behaviour and health, lack of confidence, changes to movement behaviours, changes to diet and barriers to lifestyle behaviour change.

**Conclusions:**

Our findings demonstrate that accessing behavioural and physiological feedback can increase self-awareness of how lifestyle impacts short-term health. Some participants noticed a link between the feedback presented by the two devices and changed their behaviour but many did not. Training and educational support, as well as efforts to optimize how feedback is presented to users, are needed to sustain engagement and behaviour change. Extensions of this work to involve people with diabetes are also warranted to explore whether behavioural and physiological feedback in parallel can encourage better diabetes self-management.

**Trial registration:**

ISRCTN Registry, ISRCTN17545949, 12/05/2017, prospectively registered.

**Supplementary Information:**

The online version contains supplementary material available at 10.1186/s12889-020-09740-z.

## Background

Type 2 diabetes is an increasing public health concern affecting > 400 million people globally [1] and even more people affected by prediabetes. Prevention is of paramount importance and efficacy trials have shown that programs focusing on lifestyle modification can contribute to reducing diabetes risk [2].

Several countries have implemented national diabetes prevention programs to attenuate the incidence of diabetes [3–5]. Programs in Finland and China have helped people to sustain healthy lifestyle changes to diet and physical activity for at least a year [3, 4]. Early findings from the UK’s National Diabetes Prevention Program have shown successful referral rates [5] and that the program can promote health messages in a social setting [6]. However, it is also important to consider how people at risk are supported outside of such programs, both during the programme duration (in-between sessions delivered) and after the programme ends.

The digital transformation of healthcare has grown rapidly over the past decade, with the traditional provision of medicine increasingly supported by digital tools [7]. Sitting under the broader umbrella of electronic health (eHealth) technologies, mobile health (mHealth) technologies are increasingly capable of monitoring health status and encouraging changes in lifestyle behaviours. To help encourage people to change their behaviour to promote better health, a taxonomy of behaviour change techniques (BCTs) was developed to identify “active ingredients” and improve the reporting of intervention content to ensure evidence-based techniques are employed and recorded appropriately [8]. Key techniques include feedback (such as biofeedback and haptic feedback), goal setting and self-monitoring (of behaviour and outcome). Evidence suggests that combining self-monitoring with at least one other self-regulation technique (such as goal setting) has been associated with improved intervention effects [9, 10]. Employing self-monitoring of health and behaviour in parallel aligns with the sense-making perspective [11]. Briefly, it involves evaluating new information in relation to existing understanding (perception) and, if the new information does not align with existing understanding, individuals engage in sensemaking (inference) and experimentation (action).

An important limitation to promoting behaviour change for better health outcomes has been the assumption that people are willing to make changes today to only see the benefit years or even decades later [12]. Likewise, people often pay little attention to the cumulative consequences of small, repeated decisions which in combination have a marked impact [13]. It is increasingly possible to observe the acute effect of lifestyle choices on health through technologies. However, there are several limitations to conducting research using mHealth technologies. Two key limitations often cited relate to low participant engagement in using the intervention [14] and the critical time lag where technology can become outdated by the time the research trial finishes [15]. Our aim was to explore how people at risk of type 2 diabetes engaged with real-time feedback on their physical activity and glucose levels over several weeks.

## Methods

This paper is written in accordance with the Standards for Reporting Qualitative Research (SRQR) [16] and the Consolidated Criteria for Reporting Qualitative Research (COREQ) [17]. This study was approved by the Loughborough University Ethics Advisory Committee (R17-P049).

### Context and study population

This qualitative work formed part of Sensing Interstitial Glucose to Nudge Active Lifestyles (SIGNAL) feasibility trial [18]. All participants in the SIGNAL trial consented to be contacted about an interview, using purposive sampling to ensure representation of ages, genders and group allocations. Briefly, 45 participants could access feedback from a Freestyle Libre glucose sensor and Fitbit Charge 2 activity monitor over 6 weeks. Participants were aged ≥40 years and identified as being at moderate-to-high risk of developing type 2 diabetes using the Leicester Risk Assessment Tool [19] in Leicestershire, UK. A more detailed description of how the technologies were deployed is provided.

### Intervention

Briefly, 45 participants were randomized to one of three patterns of access to feedback from the Freestyle Libre glucose sensor (Abbott, Alameda, CA) and Fitbit Charge 2 activity monitor (Fitbit Inc., San Francisco, CA), over 6 weeks. No participants withdrew from the study.

In group 1, participants could access feedback from the glucose sensor for all of the 6 weeks but were also able to access feedback from the activity monitor in the final 2 weeks. In group 2, participants could access feedback from the activity monitor for all of the 6 weeks but were also able to access feedback from the glucose sensor in the final 2 weeks. Participants in group 3 could access feedback from both the glucose sensor and activity monitor for all of the 6 weeks.

The glucose sensor communicated with a smartphone application and showed feedback relating to glucose level, direction of glucose trend, time in range and daily patterns. Participants had to scan the glucose sensor to transfer data from the sensor to the application by hovering their smartphone over it temporarily at least once every 8 h to avoid data loss. The activity monitor communicated with a smartphone application too, but also presented feedback on a wrist-worn device. The data were transferred to the application via Bluetooth. The activity monitor showed feedback relating to the number of steps taken, flights of stairs climbed, calories, distance travelled and heart rate. Brief haptic vibrations were delivered through the wrist-worn device to remind the wearer to move regularly.

### Data collection methods

Twenty-six semi-structured interviews between July–October 2017 were conducted at Loughborough University. Only the participant and researcher were present during the interviews. Interviews were scheduled to occur at the end of the 6-week intervention, taking place in evenings or the weekend. Interviews were conducted by MO and lasted < 60 min with no repeat interviews. Enrolment and interviews continued until thematic saturation was reached [20]. Transcripts were not returned to participants for comment or correction.

For reflexivity, MO is a male Postdoctoral Researcher with expertise in wearable devices in patients with long-term conditions. MO received training prior to data collection and ongoing support from CB who is a Health Psychologist with expertise conducting qualitative research. Participants were introduced to MO as a member of the study team looking to understand participant perspectives of the trial (and otherwise independent to data collection). MO informed all interview participants that his main research interests lie in people with respiratory disease but has been involved in the use of technology to support long-term condition prevention and management. No field notes were recorded.

### Data collection instruments and technologies

Interview questions were directed at revealing how participants intuitively engaged with the glucose and physical activity feedback presented by the two devices (Table 1). The schedules were initially developed by MO and CB and tested in the first couple of interviews. Interviews were audio recorded (Voice Recorder & Audio Editor smartphone application, TapMedia Ltd).

#### Topic Guide

**Table 1:** Topic guide

**Opening** Introduction Consent confirmed **Questions** How did you feel taking part in a study about your health? What were the reasons behind taking part? How did you find the devices? Can you describe how you used the wearable devices? How did you find the feedback provided by the devices? What did you think of the goals that were in place? How did you get on with the glucose feedback? How did you get on with the activity feedback? How did you find accessing the two types of feedback at the same time? How did receiving feedback make you feel? Is there anything you learned from taking part? Do you think anything has changed? What advice would you give to others using the technology? **Closing** Do you have anything to add? Do you have any further questions? **End**	

### Data processing and analysis

Interviews were transcribed verbatim by a professional transcription service. All names within the transcripts were removed and pseudonyms allocated. Qualitative software (NVivo version 11) was used for data management and to support thematic analysis. The transcripts were read and then reread by both MO and FD; this helped familiarisation with the breadth and depth of content discussed. Initial codes were then generated systematically for text that appeared relevant. After all transcripts were analysed, codes were collated into potential themes by MO and FD independently. Potential themes were discussed and reworked with the additional involvement of MW and CB until key themes were generated for the entire data-set. Names for the master and sub-themes were agreed amongst all authors to represent the essence of each theme, including choice of quotes to represent each theme.

#### Techniques to enhance trustworthiness

##### Peer debriefing

Peer de-briefing involved all authors. After the initial coding phase, the transcripts were randomly allocated to MW, CB, AK, LS and DE for coding to ensure validity, consistency and to enhance interpretive authenticity.

##### Triangulation

Triangulation and sense checking were completed through email correspondence with all of the SIGNAL participants (including those who were interviewed and participants who were not). Recipients were sent the findings of this qualitative analysis by email (namely the (sub) theme paragraphs with quotes in situ) and given the opportunity to provide feedback to the research team with any thoughts at a later date, to ensure interpretations made by the research team reflected the experiences of participants.

## Results

### Participant characteristics

Twenty-six participants (62% female, mean age 57 years) took part in the interviews (Table 2). Interview duration ranged 27–52 min.

**Table 2:** Participant characteristics stratified by risk level of developing type 2 diabetes

Pseudonym	Gender	Age range	Group allocation
**Moderate risk of developing type 2 diabetes**^a^			
Alice	Female	61–65	Group 3^*b*^
Anne	Female	46–50	Group 3
Arthur	Male	51–55	Group 2^c^
Charles	Male	51–55	Group 1^d^
Ellie	Female	46–50	Group 3
Evie	Female	51–55	Group 3
George	Male	56–60	Group 2
Isabelle	Female	66–70	Group 1
Jane	Female	36–40	Group 1
Jennifer	Female	56–60	Group 3
Joseph	Male	56–60	Group 3
Leah	Female	51–55	Group 2
Lily	Female	61–65	Group 3
Lucas	Male	51–55	Group 3
Lucy	Female	46–50	Group 2
Phoebe	Female	61–65	Group 2
Rosie	Female	41–45	Group 2
Sarah	Female	46–50	Group 3
Sophie	Female	61–65	Group 3
Steven	Male	61–65	Group 2
Theo	Male	61–65	Group 1
Thomas	Male	66–70	Group 1
**High risk of developing type 2 diabetes**^e^			
Emily	Female	56–60	Group 2
Emma	Female	61–65	Group 3
Henry	Male	51–55	Group 1
Noah	Male	71–75	Group 1

^a^Moderate risk defined as a score of 16–24 points

^b^Group 3 could access feedback from both the glucose sensor and activity monitor for all of the six weeks;

^c^Group 2: participants could access feedback from the activity monitor for all of the six weeks but were also able to access feedback from the glucose sensor in the final two weeks;

^d^Group 1: participants could access feedback from the glucose sensor for all of the six weeks but were also able to access feedback from the activity monitor in the final two weeks

^e^High risk defined as a score of 25–47 points

### Qualitative findings

Six master themes and fifteen sub-themes were developed during the thematic analysis process (see Table 3). The six master themes were (1) reasons for engagement, (2) the relationship between behaviour and physiology, (3) the various metrics shown lacked meaning, (4) changes to movement behaviours, (5), changes to diet and (6) barriers to behaviour change. Additional quotes for the various sub-themes are provided in the Supplementary Material.

**Table 3:** Master themes and sub-themes developed during the thematic analysis process

Master themes (with description)	Sub-themes
a, **Reasons for engagement** (sub-themes associated with how engagement resulted from the individual themselves, when the device delivered a notification or when other people prompted them to)	Engagement driven by the participant
	Engagement driven by the device(s)
	Engagement driven by other people
b, **The relationship between behaviour and physiology** (sub-themes associated with recognizing the relationship between behaviour and physiology, self-experimentation and the implications of this on patterns of engagement over time)	Recognizing the relationship
	Self-experimentation
	Implications over time
c, **The various metrics shown lacked meaning** (sub-themes associated with difficulty interpreting the data, feeling unsure as to how to respond to the data and seeking external sources of information)	Interpreting glucose data
	Unsure how to respond to the data
	Using online resources
d, **Changes to movement behaviours** (sub-themes associated with being more physically active and interrupting time spent sitting)	Becoming more physically active
	Interrupting sitting time
e, **Changes to diet** (sub-themes associated with making changes to the timing and type of food consumed and portion sizes)	Changing *what* food was consumed
	Changing *when* food was consumed
f, **Barriers to behaviour change** (sub-themes associated with internal and external barriers)	Internal barriers
	External barriers

#### Reasons for engagement

The driving factors for engaging with the devices differed between participants. For some, their engagement was mostly driven by the technologies reminding them or prompting them to engage (e.g. vibration prompts were received or notifications delivered). Others were more proactive in engaging with devices (e.g. at particular times of the day). There were also socially-driven reasons for participants engaging with the technology, such as actively showing people the glucose sensor or other people noticing it and asking participants to explain what it was.

##### Engagement driven by the participant

Participants most often reported scanning the glucose sensor around times of eating as they felt this pattern of scanning could show the effect food was having on their glucose levels.

“I just used to start looking at it whenever I had anything to eat” (Isabelle)

Glucose sensor scanning was also often done at particular times of the day to help them remember to scan the glucose sensor.

“I like structure and it helps me to remember things so I had to do it in the morning, I did it at lunch time and around 6 o’clock” (Sophie)

Other participants reported scanning more regularly throughout the day and several participants reported scanning when they remembered. In comparison, participants rarely mentioned looking at physical activity feedback around specific events. Instead, participants entered information about bouts of non-ambulatory exercise via the Fitbit as these activities were not automatically detected and developed a routine for charging the activity monitor.

“I have done swimming, obviously the Fitbit doesn’t measure the swimming, so I have logged all that” (Rosie)

##### Engagement driven by the device(s)

Haptic feedback from the activity monitor, in the form of a gentle vibration, encouraged participants to move by raising awareness of time spent sitting. For some, the haptic prompt caused a positive reaction.

“For it to buzz and say do another fifty and you think god have I only done two hundred steps, it raises your awareness definitely” (Evie)

The glucose feedback application, in comparison, delivered notifications to remind participants to scan the glucose sensor and this often triggered engagement.

“If it reminded me I would do it there and then” (Alice)

##### Engagement driven by other people

Several participants recognized how monitoring glucose levels could be a valuable tool for people living with diabetes, commenting on how they spoke with other people who might benefit*.*

“He [colleague with diabetes] said if that comes out on the NHS then it’ll save him pricking his finger three or four times a day which he does at the moment” (George)

Conversations around the activity monitor most often involved comparing numbers for the metrics relating to how much (or how little) they had done. Several participants described how they showed the glucose sensor to others*.* Heightened attention and interest may have been due to the visibility of the glucose sensor on the upper arm compared to a wrist worn device. The feedback presented by the glucose smartphone application was also of interest, with the glucose levels entertaining family members. They also discussed how other people found scanning the glucose sensor novel and how family found watching them do it entertaining.

“The family were laughing the first day I put it on and after we’d eat, it was going up and up and up” (Lucas)

#### The relationship between behaviour and physiology

Participants described how they could see the effect of their diet and physical activity on their glucose, with some people deliberately investigating how certain foods or activities influence their numbers. As they went through the study and became more familiar with these relationships or no longer saw value in seeing the information, they did not need to look at their data as often.

##### Recognising the relationship

Many participants recognized a relationship between the food consumed and the immediate resulting effect on their glucose levels. This may explain the tendency to scan around mealtimes or times of eating. Participants also identified that peaks in glucose levels were often consistent in frequency and timing around main meals. Interestingly, participants noticed how different foods had varying impacts on glucose, in particular how fruit caused immediate increases whereas alcohol and chocolate did not.

“In my own mind, I wanted to get from this whether I should give up chocolate and stuff but then that wasn’t really having a huge effect. It’s the fruit” (Emma)

Fewer participants identified a relationship between physical activity and glucose levels. Participants identified the relationship between diet and glucose more clearly, whilst some found it difficult to make inferences of the two forms of feedback. However, participants found that being physically active brought their glucose levels down quicker than no activity.

“One thing I’ve noticed is if I eat and I’m not active afterwards, the glucose goes higher than if I eat and I take the dog out, even for ten minutes. It seems to make a difference” (Thomas)

Some participants further described how the intensity of physical activity also impacted glucose levels.

“I also noticed after the evening meals or any meal, if I did some exercise it helped to bring it down more quickly and stayed down. But if you overdo it, it goes up again” (Jennifer)

##### Self-experimentation

Several participants spoke about how they experimented with the amount and type of particular foods they ate to see how this influenced their glucose levels. Some participants were less specific in describing how they experimented with their lifestyle behaviours, but they often changed what they ate or what they did afterwards.

“I’ve seen the three daily bumps … That gives me a little clue about how to deal with them, if those bumps get too high, in terms of what I put into what I eat or what I do afterwards” (Thomas)

Some participants discussed how they had experimented to see the impact any changes had, whilst some participants described how they did not even consider experimenting with their diet and activity*.*

“I think I might have planned better what I was going to do … I should have been a bit more scientific about it.” (Jennifer)

##### Implications over time

Several participants explained a reduction in frequency of scanning the glucose sensor because the glucose feedback became familiar, recognizing how glucose levels were impacted by behaviour.

“Because I didn’t need to. I knew what the pattern would be pretty much. And I was more or less right” (Rosie)

Despite engaging a lot with the glucose sensor at the beginning, many experienced a loss of interest over time, with several noting how they almost forgot they were wearing the glucose sensor. It is possible this loss of interest was because they didn’t find personal value in seeing the numbers.

“I think the only reason is because I’m not diabetic so it wasn’t something that was on my mind and I had to do it” (Evie)

Similarly, engagement with the activity monitor was initially high before eventually avoiding the vibration prompts to move. Reasons for loss of interest were in part because they knew how inactive they were and did not need telling and found other physical activity features more interesting.

“I was constantly checking how many steps I had done... It was kind of the first couple of weeks, I was glued on it” (Lucy)

#### The various metrics shown lacked meaning

Difficulties in making sense of the data provided by the glucose sensor and, in particular, what to do about it, were reported by participants. This was less of an issue for the physical activity data. This drove some participants to seek further guidance online with mixed success.

##### Interpreting glucose data

Participants described how they did not know what the glucose data meant in relation to the target zones. Others reflected on how they could not understand why glucose fluctuated.

“I’m not sure how high it should go or shouldn’t go and whether it’s okay or not” (Jane)

In an effort to assign meaning to the glucose feedback, some participants paid attention to how much time their glucose levels spent within the normal range (green zone). In comparison, others tried to reflect on behaviour, and as a result of not seeing a need to make any changes, didn’t change what they ate.

“That was when I thought perhaps I should check out what I’m doing and not doing. I still don’t know what was causing it, so I couldn’t work it out” (Emma)

##### Unsure how to respond to the data

Several participants described feeling uncomfortable making changes to their diet whilst not understanding the meaning of their glucose levels. Many could also not figure out how to change their heart rate which was monitored by the Fitbit.

“I am not going to start messing round my diet for the sake of something I don’t understand” (Henry)

##### Using online resources

Several participants looked online to understand their glucose levels with some reflecting on what they felt when they found out what the number should be.

“I did Google it, so I got some information on that. It seems to suggest that that number was okay” (Lucas)

For some, researching online did not bring any new information or meaning.

“I did a little bit of basic research to understand what I was doing and should it be going up and down like this but I didn’t really find out very much” (Sophie)

#### Changes to movement behaviours

Participants cited the technology as an important driver to move more and sit less and with more emphasis on the usefulness of the activity monitor features than the glucose sensor features.

##### Becoming more physically active

Participants described how they were generally more physically active during the study because the feedback from the Fitbit encouraged small changes. Increases in physical activity were often attributed to using the Fitbit’s activity feedback metrics, including the 10,000 step count goal and 250 step hourly goal.

“It did encourage me to walk more, I must admit. It kept buzzing asking me to do another fifty steps “ (Evie)

The Fitbit activity monitor gave rewards if particular goals or milestones were reached. Participants welcomed this form of positive reinforcement whilst others made their decision depending on how many steps were needed. Motivation to reach the Fitbit’s step goal was often driven by the reward of relaxing afterward.

“I just stroll up and down and then it all goes … and you think, ‘that’s it, I can go and sit down now and watch the telly’” (Phoebe)

##### Interrupting sitting time

Some participants identified being more aware of time spent sitting, expressing surprise at how much time they spent sitting after seeing feedback from the Fitbit. This awareness of extended sitting periods was often attributed to the activity monitor’s “reminders to move” feature.

“The Fitbit did show me I spend a lot of time sitting on the couch, surprisingly more than I thought I did” (Sophie)

The Fitbit hourly vibration alerts gave participants an opportunity to act on the information in a timely manner. For others, these prompts were felt to be a nuisance.

“They [Fitbit vibration prompts] just irritated me” (Sophie)

#### Changes to diet

Participants cited the glucose sensor as an important driver to improve their dietary behaviours, specifically what food they ate and when.

##### Changing what food was consumed

Some participants changed what they ate after seeing prominent spikes in glucose via the Freestyle Libre. One participant outlined how they previously tried to stop eating biscuits unsuccessfully, but the glucose sensor provided physiological evidence to make the change.

“I’ve tried that [not eating biscuits] before, using my own willpower but with the power of this, it has made it a lot easier, because the evidence is there” (Thomas)

Others described how seeing the impact of particular foods on glucose made participants realize consuming healthier foods might not result in an improved immediate glycaemic response. Participants reported how observing improvements in their glucose levels following small changes encouraged them.

“Over 14 days, I could see, for instance, it would tell me that all my average levels were orange every time. Whereas if I looked at it for just 7 days at one point it was green, so I could see that my actions had made a difference” (Rosie)

##### Changing when food was consumed

Participants reported making changes to the timing of eating particular foods as a result of monitoring their glucose levels. Several participants noted the need to spread their consumption of carbohydrates throughout the day.

“I am trying to sort of have a bit of carbohydrate with each meal, rather than trying to go without all day and just have it in the evening” (Leah)

Others reported having a better awareness of when to stop eating as a conscious decision given feedback presented by the Freestyle Libre.

“I wouldn’t say I stopped overeating and things but it made me much more aware of consumption and stopping when you’re full” (Ellie)

#### Barriers to behaviour change

A range of internal (e.g. comorbidities) and external (e.g. weather) barriers to behaviour change were noted by participants.

##### Internal barriers

Several participants felt they were already physically active and this limited their motivation to do more. In contrast, some reported comorbidities and generally feeling unwell as key barriers to increasing physical activity. Some participants concluded their glucose data as not being a concern.

“It would give me an insight into what was happening in the body which to my knowledge it followed the path that it should follow” (Noah)

Participants disclosed that if the glucose levels had been elevated for longer, it may have prompted a reaction.

“It is a spike, it’s not like it goes up and it stays up. I think that would have induced panic” (Sophie)

##### External barriers

The Fitbit vibration prompts to move were often experienced whilst working on a particular task or in a meeting. Despite finding this prompt helpful to raise self-awareness of time spent sitting, participants often felt they were unable to respond to them at work.

“Half the time I’m engrossed in doing something or if I’m trying to get something done, it’s a bit of a distraction getting up and walking off” (Lucas)

Rain and cold temperatures were also typically reported as hindrances. As well as the physical challenges in daily life, several participants reported how societal norms impacted their ability to respond to these prompts.

“If I was at friend’s house or we had a friend at our house, you can’t really get up and walk around can you?” (Noah)

Caring duties were also mentioned in the context of children and family members.

## Discussion

This study explored the perspectives of people at risk of developing type 2 diabetes receiving feedback relating to health and behaviour in a real-world environment. Our findings revealed that participants were driven to engage with the two devices either by themselves, device notifications or other people. Some participants could recognise a relationship between their behaviour and their glucose levels and behaviour change resulted. However, comments were raised that the data shown lacked meaning for several participants and there were barriers to making changes to diet and physical activity levels.

Participants made changes to their diet and physical activity levels as a result of recognizing the link between behaviour and physiology; driven primarily by the feedback provided by the glucose sensor rather than the activity monitor. This suggests that having access to physiological feedback can raise self-awareness and deepen understanding of how the body functions. It appears possible for people to interpret how their behavioural choices, such as going for a walk, immediately impact glucose levels and there did not appear to be any gender differences as to whether people recognised this effect. Real-time access to glucose levels may act as a silent persuader to encourage positive behavioural choices [21]. The notion of seeing how glucose or blood pressure can vary in relation to other behaviours, including diet and exercise and extending to medication, has been observed elsewhere [22–25]. Similar to present observations, studies involving self-monitoring of blood glucose have shown potential for people cutting down on sugary foods [24], increasing activity levels [23, 24], and improving medication adherence [25, 26]. Being able to understand physiological data in the context of wider factors can help people assign meaning to the feedback [27] to supplement specific physical activity feedback. Participants recounted how going for an after-dinner walk lowered their glucose faster than if they were sedentary and this observation was not facilitated by feedback from the activity monitor.

Participants naturally engaged with the glucose sensor around mealtimes or at set times of the day, facilitating the recognition of the relationship for some. This aligns with the importance of the frequency and timing of self-monitoring blood glucose levels [28, 29]. However, some participants structured their engagement around avoiding data loss, bringing into question whether they would have engaged had this requirement not been in place. However, no gender differences were recorded. The variability in the approaches taken by participants demonstrates the importance of encouraging scans around opportunities to learn about physiological responses to behaviours. Scanning was also mentioned in the context of showing other people, reflecting findings of a text messaging intervention where people openly shared the messages with family members [30].

The novelty effect of having access to new technologies supports existing phenomena; however, there were contrasting reasons behind this observed reduction in usage. Some participants became more efficient with interpreting the data or how they did not need to look at data as frequently because they become increasingly aware of bodily symptoms and signs [28, 31]. Our findings emphasize the importance of understanding the reasons why some people use these technologies less frequently. It is worth noting here also that a reduction in use over time may be because our sample comprised people at high-risk, rather than people with a diagnosis of type 2 diabetes. Targeting high-risk populations can have implications. One in particular is that the information provided by such technologies may not show sufficient health risk so, despite being categorised as being at high-risk, as things stand their physiological parameters may be healthy. Maintaining normal physiological health is paramount but this information may not be motivational to make changes if no changes are visibly required. Another implication relates to the cost of targeting at high-risk groups; namely, the number of people living at high-risk far exceeds the number of people living with the condition [32] and so an economic assessment would be needed to confirm a return on investment.

The reduction in usage reflected that many participants were unable to respond to the feedback being presented. Studies have previously described how people with diabetes often find glucose levels challenging to interpret [31], and how more than half do not know what action to take [24]. Digital health technologies may be appropriately placed to offer support during such events. However, data alone are unlikely to be sufficient. There was confusion caused by misleading insights into the immediate health effects of chocolate versus fruits. People could be misled into thinking less healthy foods might be better because they cause a better acute glucose response [22, 31]. The off-the-shelf deployment strategy has identified a need for additional information or training beyond what is provided by the technologies.

Several participants discussed increasing their physical activity, interrupting sitting time and making changes to when and what food was consumed. This may be in part because participants found the glucose feedback motivating to make changes [33, 34]. It could also be because the two devices provided feedback that was actionable and continuously available [35] and offered information on the health consequences of behaviour [36]. However, barriers to behaviour change were notable. Participants found that living with comorbidities, societal norms and weather restricted an opportunity to change their behaviour. This is not uncommon, with the wider literature citing barriers around health problems, lack of time and weather [37], as well as coexistence of other poor lifestyle behaviours and misinterpretation of messages as barriers to behaviour change [38]. With continued technological advances, it is increasingly feasible to overcome some of these barriers. For instance, taking into account the context of the person, integrated smartphone sensors could deliver notifications at times of the day where the weather is acceptable or when they are not in a work meeting to create a more receptive environment for behaviour change.

The small proportion of participants with prediabetes limited comparisons with at-risk individuals and recruiting people through community approaches may limit generalizability. Multiple interviews could have provided greater insight into what it was like for participants to use the technologies. Our findings are limited to short-term engagement with digital health technologies and would benefit from a longer duration of access.

## Conclusions

This study suggests that receiving feedback on behaviour and physiology can increase awareness of how lifestyle choices impact short-term health. A proportion of people can intuitively notice a relationship, with some deciding to make changes to their activity and food consumption. Supplementing these technologies with training and educational support is needed as is future research to optimize feedback on physiology and behaviour. Extensions of this work to involve people with diabetes are also warranted to promote better diabetes self-management.

## Supplementary Information

**Additional file 1.**

## Data Availability

The datasets generated and/or analysed during the current study are not publicly available due to them containing information that could compromise participant privacy/consent but are available from the corresponding author on reasonable request.

## Abbreviations

COREQ: Consolidated Criteria for Reporting Qualitative Research
mHealth: Mobile health
SIGNAL: Sensing Interstitial Glucose to Nudge Active Lifestyles
SRQR: Standards for Reporting Qualitative Research
UK: United Kingdom
