# Supplementary material for: A digital lifestyle behaviour change intervention for the prevention of type 2 diabetes: a qualitative study exploring intuitive engagement with real-time glucose and physical activity feedback
Source: BMC Public Health. 2021 Jan 12;21:130. doi: 10.1186/s12889-020-09740-z (PMC7805160; doi:10.1186/s12889-020-09740-z)
Supplement: Supplementary file 1 — Additional file 1. [file 12889_2020_9740_MOESM1_ESM.docx]

**SUPPLEMENTARY MATERIAL**

**Additional quotes**

REASONS FOR ENGAGEMENT

ENGAGEMENT DRIVEN BY THE PARTICIPANT

*"I tried to sort of have a bit of a thing where I did it perhaps every couple of hours or something like that so I'd get up and do it " (Sarah)*

*“Just whenever I thought about it, there wasn't any regular pattern to it" (Anne)*

*"Just when I could remember because I’ve got a horrendous memory" (Emma)*

*“I did get into a routine of taking the Fitbit off and charging it up in the evenings" (Sophie)*

*"Pre-breakfast and then after, it showed what it peaked and then came down"* (Noah)

*"I think I’d sort of scan it before eating and after to see what affect it had and what it looked like, so yeah, on occasion I would try to see, say a meal or if I had some biscuits to see what affect it had."* (Jane)

*"I scan when I first wake up, before I eat and approximately two hours after breakfast, before I had a coffee in the morning. I scanned, similarly, two hours after lunch and last thing at night and if I got up for a pee, I scanned in the middle of the night. They were the regulars"* (Thomas)

*"I just did it at random because I know it’s supposed to remind me but I never noticed the reminders. When I remembered, I did it"* (Joseph)

*"I did find actually the timing, you know because you have to scan it within eight hours each time, I got myself into a little pattern. I did it four times a day because I was a bit worried about missing"* (Sophie)

ENGAGEMENT DRIVEN BY THE DEVICE(S)

*"I just automatically put it on, just worked like a watch really, didn’t think about it and then as it buzzed, you know okay, what have I got to do now?" (Emily)*

*"The little 250 step things would make me look at it and a couple of times I would think, ‘right, I will do my 144 steps’"* (Ellie)

*"No, it’s kind of like ‘have a nice day’. It’s not me anyway, that’s for sure. It doesn’t work for me at all, no. It’s like nagging and I don’t want to be nagged"* (Alice)

ENGAGEMENT DRIVEN BY OTHER PEOPLE

*“My husband certainly said oh I could do with one of them, I'd be interested to know what I do… so it does spark conversation as well" (Anne)*

*"When I said it was a glucose monitor she [lady with diabetes] said I’m wanting one of those [glucose sensor], so then I got the phone out and I was showing her" (Emily)*

*“Some friends saw it because obviously sometimes you could see the disc through clothes and they would ask me what’s that" (Lucy)*

*"Days when it’s being checked at ten-minute intervals it’s probably where I’ve been with [my niece and nephew], with them going, ‘can I do it now? It’s my turn to have a go’" (Ellie)*

*"My husband thought it was hilarious that at certain times of the day I would pull out the phone to my arm" (Sophie)*

*"[my mother] was quite fascinated to see what did that translate to, I mean she doesn't do kilometers obviously, but distance-wise we were trying to work out what 2,000 steps was supposed to be… which she could relate to"* (Sarah)

*"Yeah, she’s not in my office but I had a quick chat with her and she was telling me about she basically uses it all the time but she can’t control it and she said it’s been really helpful for her to see sort of real time. She doesn’t have the app, actually, she has one of these readers which I think the functionality isn’t as good, she can’t do as many things with that and so, she was quite excited like oh, I didn’t know you could do that… She was saying that 70%, oh I hope for that, because she was all over the place. I had a quick chat with her"* (Jane)

*"Everybody was interested. In fact, one of my colleagues is a diabetic and he was very interested in scanning it rather than, he has to prick his finger and check his blood like that"* (Ellie)

*"If you’re going along to your doctor with that app … I showed it to my doctor last Friday. He’d never seen one, he was fascinated. He looked at it and said, ‘there’s no danger of you ever getting diabetes, is there, because it’s never gone out of the green’"* (Phoebe)

*"They see the reading going on. I think my friends were split into two camps, really. The techie ones who were interested and all over it and those that just thought the whole thing was just a complete bore"* (Theo)

*"The two kids were fascinated as well. I was showing them my blood sugar level and then they ended up doing it, just put the phone next to it. It is very clever"* (Evie)

THE RELATIONSHIP BETWEEN BEHAVIOUR AND PHYSIOLOGY

RECOGNISING THE RELATIONSHIP BETWEEN BEHAVIOUR AND PHYSIOLOGY

*"Have something to eat and it goes up" (Steven)*

*"Sometimes I’d check to see what happened on seven days, fourteen days, 30 days, just to see. I found that the peaks were almost always in the same place" (Alice)*

*"I was amazed how much bananas altered it… I ate a banana and then a few minutes later I would have a bit of a peak” (Joseph)*

*"I was most impressed by the fact that I could see the effect of what I was eating and that was more interesting than the exercise thing" (Charles)*

*"To see the difference, to see visibly once you've eaten and then it remains high unless you do some exercise and then once you do some exercise, you see it go down, it's really amazed me" (Anne)*

*"During the day I get it, you can have a meal and suddenly there is a spike... And if you wanted to educate somebody about their diet, you can say, well this is what happens"* (Henry)

*"Yet, it doesn’t go up when you drink alcohol. I would have thought it would have gone ‘voom’ but it doesn’t. How interesting is that?"* (Phoebe)

*"I think I noticed when I had carbs, obviously that went up quite high then, with a meal with rice or potatoes or that sort of thing"* (Jane)

*"On the last day, it seemed to go right up. (That one was the worst ones, although I suppose I would have had breakfast at that point) I tended to have porridge for breakfast, which I thought was good, but with fruit. I think it was perhaps the fruit that was doing it"* (Emma)

*"It dropped really quickly after a period of physical, sustained physical activity, it dropped really quick, literally down to 3.8, so getting to the red zone"* (George)

*"What was interesting was seeing last week, when I was able to go jogging properly on the treadmills and my blood sugar levels went up when I was jogging. There must be something happening in my body to release it"* (Joseph)

*"Every day I see three quite distinct bumps. I see a bump after my porridge in the morning, I see a bump after lunch and I see a bigger bump after dinner. If I have a drink, there’s a few little ripples going on towards midnight. If I’ve done something active after one of those bumps, if I go in the garden after lunch, say, I notice, not necessarily that the bump doesn’t go so high but it comes down more quickly"* (Thomas)

*"if you don't sit for too long after a meal your blood sugar will come down much more quickly, even if you don't go for a walk"* (Jennifer).

*"it shows what you’re putting into your body in quite a real-time way, what effects certain things are having"* (Ellie).

*"it just seemed to me it would perhaps be better to link it to the food I was eating and the exercise rather than just the exercise because obviously that is going to make a difference, bearing in mind that big spike. Honestly you eat a bar of caramel chocolate and you get a huge spike, it’s not really rocket science"* (Sophie)

SELF-EXPERIMENTING TO UNDERSTAND THE RELATIONSHIP

*"I experimented with the quantity of cereal, that was it. If I had one Weetabix biscuit was it completely different and it was less" (Ellie)*

*"Just showing them the graph, showing them what kind of strategies I’d adopted, what worked and what didn’t" (Theo)*

*"So, I would want to change my diet more. So, for instance, change to different things I would drink in the morning, see how that would affect it. Or to see whether I ate something fibre first, then have the drink, whether that would smooth it out a bit. And I haven’t had enough time to be able to do that"* (Rosie)

IMPLICATIONS ON ENGAGEMENT OVER TIME

*“I checked my glucose levels for four weeks and I knew if I had have eaten something really naughty it was going to go up and I knew it was going to come back down” (Evie)*

*"To start with I was obsessed. Like, measuring it all the time. Because I was just absolutely intrigued” (Rosie)*

*“On the third one I had almost forgotten about it” (Evie)*

*"Took notice for maybe a week or so” (Rosie)*

*"I just found myself checking it… more the thing on your arm than actually the Fitbit thing because I know I'm lazy so it wasn't telling me anything I didn't know already" (Sarah)*

*"The most interesting bit for me was my heart rate, not the activity" (Rosie)*

*"The only thing was the last couple of weeks I forgot I had it on and I didn’t scan as much. I didn’t do as much of everything and I don’t know and I don’t know why. Whether it was because I had either become complacent with it or whether I just forgot it was there… No, strangely enough it didn’t, I think it was because I checked my glucose levels for four weeks and I knew if I had have eaten something really naughty it was going to go up and I knew it was going to come back down and I knew it was low in the morning. It was almost like nothing was changing with that, but with the Fitbit you could change it and improve with it. That’s the only reason I can think of. I did scan over the last two weeks, and probably over the last two days I have done it but not at the frequency that I started, like I said earlier. I did become quite complacent with that but not with the Fitbit"* (Evie)

*"When it was first in, I was scanning all the time because it was a novelty and then after a few weeks, you tend to forget it’s there"* (Joseph)

*"It’s like a new toy, you kind of think, let’s use it to monitor. It was prompting me to walk 250 steps per hour and I was thinking, off I go, up the stairs and counting how many stairs I had done. I was constantly checking how many steps I had done, how many stairs I had climbed and how much activity I had done. It was kind of the first couple of weeks, I was glued on it. It would probably show on my report I was really active and then show I wasn’t doing anything now"* (Lucy)

THE VARIOUS METRICS SHOWN LACK MEANING

DIFFICULTY IN INTERPRETING GLUCOSE DATA

*"But I just couldn’t just work out why suddenly it just shot up, then other times it was really low" (Lily)*

*"I wouldn’t want to be less than two-thirds in the green band. I don’t know why but I felt that was the right thing to be " (Alice)*

*“it’s all pretty color. That was it really. I couldn’t identify what put me up or what put me down" (Lily)*

*"Although, when I had chocolate, my blood sugars used to go down quite often, which was a bit odd. I couldn’t work out what I should be eating and what I shouldn’t but it did make me think about it"* (Emma)

*"I still don’t know whether my glucose level is higher than it ought to be. It seemed to be within reasonable bounds, when you look at the long-term trends but the curves seemed to be within the ‘sweet spot’ most of the time but it does go significantly above it sometimes"* (Theo)

*"I don’t think I ever went out of the top level, was that 9 or something? A good percentage of the time I thought I was within the level. I didn’t understand that going below the level, was that 4, I think the band was 4-6. I often didn’t really understand why I often went below that but I did briefly a few times and I never really knew why that happened so I didn’t understand that but I was still fascinated"* (Isabelle)

*"It was just willy nilly. Sometimes it was up, other times it wasn’t, most times it was normal, then something shot up and I think, Oh I don’t know what I did different to make it go up there. But I have got quite into sort of dobbing [scanning] and testing it"* (Lily)

*"No, I don’t think it worried me too much because I knew there was nothing I could do. I couldn’t actually pinpoint when it had happened. Perhaps you can pinpoint it but it didn’t seem obvious to me that the blip had happened at that exact moment"* (Emma)

UNSURE HOW TO RESPOND TO THE DATA

*"My heart rate’s quite high at times and I was just monitoring it. I couldn’t think of anything to do to reduce it" (Theo)*

*"It was a bit scary to be honest because I have the same breakfast every day. I don’t tend to have lunch and I eat quite late in the evening as well. Then for some reason it decided to go up in the night, which was most peculiar. Sometimes it went down, sometimes it went up"* (Alice)

*"When it did go up I suddenly thought, ‘actually, I am bothered’ because it’s like ‘what have I done, what’s happened?’"* (Emma)

*"I would probably panic a bit really. But as long as there was something to reassure me that’s it’s OK for that to happen. But obviously if it’s gone into the danger zone, I would need to know what to do about it really. But as long as it’s sort of explained that it’s safe, that’s it’s not too abnormal. I suppose if it kept happening I would probably go and see a Doctor and get it investigated"* (Leah)

USE OF ONLINE RESOURCES

*“It will estimate your A1C and then, you know, using Dr. Google, you can find out what it should be, and then you know, make you feel even worse about yourself" (Rosie)*

*"It was quite interesting to think should I really find out more about it? I have no idea if that’s normal, is it supposed to go up and down the way it does. That is something I may well have a look at"* (Sophie)

*"I did have a dabble trying to find out how many steps I should be doing but I didn’t find anybody who seemed to think it should be any different, perhaps it shouldn’t, so I soon gave up on them and thought I would stick with the 10,000"* (Isabelle)

*"I put the last one on right round here because I did have a look on the diabetic website and it did say not to put in in muscle, it worked better when it’s in the flappy bit of your arm, so that’s what I did the last few weeks because I did it before on this bit so then I put it further round as it shows you"* (Emily)

CHANGES TO MOVEMENT BEHAVIOURS

BECOMING MORE PHYSICALLY ACTIVE

*"If I had a sort of half hour spare and it wasn’t very high I would go out. It has made me conscious of walking more, try to not use the car so much" (Leah)*

*"I just like looking at it and seeing how many steps I have done. When you get to 10,000 there is little fireworks" (Leah)*

*"I would go out at midnight just to get to when you get your stars and the rocket goes off" (Phoebe)*

*"It will say you only need to do 200 more steps and you’re there. That was encouraging because you think, I’m not going to fail at this stage" (Isabelle)*

*"I am definitely walking further and more. Not a huge amount, but certainly more than I was. It does highlight it to you, you’re thinking oh fifty steps right I can do fifty steps, it’s just a little trot around the corner"* (Evie)

*"Then we had some fun because I said, “Oh, I haven't done my 10,000 we have got to go out for a walk”, so we all went out for a walk around the square and stuff like that. It was quite good fun… I think we definitely walked more"* (Jennifer)

*"The vibrating thing was good. I did like that every now and again telling me to get up and do something. I did try and do that as well. I think most days I probably did my ‘nine of nine’. I quite liked the funny little remarks and things it said"* (Emma)

*"I forced my son to go out for a walk on a couple of evenings which was good because it was during the summer holidays and over the summer so it was easy to go for a walk round the estate and see how many steps I'd done which again is something I wouldn't have normally have done so it did make me do it just to gain a few extra steps"* (Sarah)

*"On a couple of occasions I went up and down some stairs a couple of times to make sure I hit my ten flights of stairs in a day"* (Sophie)

*"It feels good. I know it’s not an Olympic gold medal but nevertheless, it’s something. I know how easy it is to be inactive. I know there are people my age who are very inactive and I’ve always prided myself on being able to move around and do stuff. I quite enjoy feeling energetic. Having [the Fitbit] there has motivated me and it’s confirmed and affirmed the efforts I’ve made. You get the little pyrotechnic displays, ‘boom’, which helps… I used to look forward to that"* (Thomas)

*"I did try and achieve all of the goals for all of the things. I thought, ‘that one down’. Because I was quite close to it, I wanted to see if I can get them all because I’m quite close. It does give you a bit of enjoyment, that you’ve met the targets"* (Lucas)

INTERRUPTING SITTING TIME

*"I’d better get up. I’ve been sat at my desk for an hour’. It did make me get up and walk" (Emma)*

*"Yes. I think it’s about ten to the hour it does it. When you have not done enough in the hour. So, what I did a couple of times, I would go up and down the stairs a few times to sort of do it. If there was a break in the work we were doing. That was quite handy"* (Leah)

*"It was great. Yes, one reinforced the other really. If it showed that I was doing a lot of it, if the Fitbit shows I was doing a lot of exercise and I could see on the tracker where my sugar levels were going down so I could see why I was feeling a bit funny after fitness and that and it explained that to me"* (George)

*"I do, yeah, I do or I walk up and down the garden because it's about 200 steps up and down the garden. I will try and move, yeah. Especially when I know I haven't moved"* (Jennifer)

*"When I was aware of it I thought, I’ve got a one hundred and fifty more to do, or whatever the case was and I would go and do it, or try to"* (Noah)

*"It was alright. It did encourage me to do a few more steps a few days to try and hit the target and get your celebration rewards at the end. It did help a little bit, it gives you a jolt every hour to get you to do 250 steps. It does help sometimes but it does depend at the time whether you can fit it in or not. It did seem quite good actually, it did seem to encourage a bit more activity... Just a quick walk round the block in the office and you can get your steps up doing that"* (Lucas)

*"Even though I was interested in this [reminders to move feature] and even though I used it, I didn’t use it as a carrot and stick kind of thing"* (Theo)

*"I did what it told me to do really, and it encouraged me to do more, without a shadow of a doubt"* (Evie)

*"It sort of pushed me to do it if I had time to go… Yeah, it sort of just nudges you, get out of your chair sort of thing… It’s sort of just like a little reminder. Stop you being a couch potato. It seemed to help"* (Leah)

CHANGES TO DIET

CHANGES TO *WHAT* FOOD WAS CONSUMED

*"I’ve cut out biscuits because I can see that I don’t need a biscuit” (Thomas)*

*"I think if you have the evidence in front of you, you can't deny it" (Anne).*

*“I was stunned that that [white rice] spiked above the McDonalds… I was really quite amazed by that kind of thing and I suppose that’s what got me into checking it more and more because I thought hang on a minute I think I'm being healthier and actually from a blood sugary kind of thing it spiked, it spiked more" (Sarah)*

*"I didn’t like seeing big spikes in the graph, so I cut down on chocolate"* (Ellie)

*"Well it was noticing how high it went with what I was consuming really so yeah whether it be food or drink. So, I found myself consciously avoiding high sugary, I don’t really have a sweet tooth, but high sugary type things, the banana being the most surprising and breakfast cereals. I am drifting off completely breakfast cereals, they are just too high in sugar I think"* (Charles)

*“I used to eat way too much chocolate. I haven't had chocolate since I started the study, I've cut that out, and one or two packs of crisps a week rather than one or two a day"* (George)

*"So, yes, it's just made me look when I'm in the supermarket let's see what the alternatives are, see if that works any different to having rice with my meal or something like that"* (Sarah)

*"I wouldn’t say cut it out, I would say less frequently. And I probably, I mean I have always drunk, apart from orange juice to be fair I always drink water and sugar-free drinks. I don’t drink tea or coffee. But I guess it has made me make a more active decision to do that. So sometimes maybe when I got home from work, I might have a glass of juice. Now I won’t do that because I know it will spike. So, it definitely has made me think more carefully"* (Rosie)

*"I did cut down on sugar, so I stopped having it in tea"* (Ellie)

*"My wife had said when I had done the first reading that was high, well don’t have any sugar in your tea and I replied, I like sugar in my tea. I did consciously cut down from a full teaspoon to slightly less"* (Steven)

*"I've cut down on my sugar intake. I've stopped taking sugar in tea, I use a sweetener thing now so that's going to help with the higher levels hopefully"* (George)

CHANGES TO *WHEN* FOOD WAS CONSUMED

*"I’ve got accustomed to eating too much, full stop. For a 65-year-old man to say that is a piece of new information is ridiculous really but nevertheless, it is new information"* (Theo)

*"I thought, ‘well I’m getting these quite big spikes from having lunch’, which would normally be a bread roll with something, salad and fruit, would be my usual thing. The fruit was making it … so I thought maybe I should have salad and a roll at lunchtime and then in the afternoon, instead of tea, I’ll have my fruit then and it’ll lead to a more stable kind of thing. Often, I’ve thought well at lunchtime I’m just having my normal lunch, not because I’m still hungry after that but because it’s there and because that’s what I normally have. So, I did change that"* (Ellie)

BARRIERS TO BEHAVIOUR CHANGE

INTERNAL BARRIERS TO BEHAVIOUR CHANGE

*"I will get the steps up if I go out. I have always walked round the long way" (Leah)*

*"You know when you can climb up the side of a hill and stuff without having to stop every two minutes, you know that you're okay" (Jennifer)*

*"I’m not 100%… This has all happened since I had pneumonia in February, which has meant I don’t exercise as much " (Evie)*

*"It looks like the majority of the time I’m within the band, just going in and out sometimes when I’m eating and stuff like that. It seems okay, to be honest, from what I’ve seen"* (Lucas)

*"I think to be honest I think because it was monitoring and I was normal, it’s not scared me but if it showed I was high in sugar I think it might have made me think oh my god, you need to stop eating that cake or stop having so much sugar. The times that I have monitored it, it has been quite constant which meant it hasn’t scared me yet"* (Lucy)

*"It all seemed okay to be honest, nothing alarming in there or anything. Nothing that sets alarm bells ringing, thinking perhaps I’ve got diabetes or anything like that"* (Lucas)

*"I have not changed one thing to what I was doing before to what I am doing now. Not one thing. I have not walked any further. I have not ate any different foods. Tell you what, I have not changed my diet. I have done nothing different… It hasn’t alerted me to anything in particular"* (Lily)

*"I do generally cycle to work, so I get a bit of exercise doing that anyway"* (Lucas)

*"Over the last couple of years, I’ve an activity tracker like this, although I don’t do an awful lot of exercise of various sorts in the main I try to run most days, a short run, because I’m exhausted it tends to be two kilometres and that’s been going on, although I’ve been using activity tracker for a couple of years, it’s probably in the last nine, ten months that I’ve actually been running"* (Arthur)

*"When I first had it, I had a bad ankle so I couldn’t do much walking. I have been away twice from I had it so my lifestyle has not been sort of normal. Not doing what I normally do"* (Leah)

*"Some days I can’t walk very far because me back’s terrible [because of osteoporosis]"* (Phoebe)

*"My health has gone downhill and I have been to the hospital in the evenings to visit her [daughter]. So, I haven’t had time to exercise. So, it really has came at a bad time... Yeah. Because I have not really, well you have been able to see my numbers and maybe I have not moved up at that side”* (Rosie)

EXTERNAL BARRIERS TO BEHAVIOUR CHANGE

*"I’m not going to go for a walk in the rain" (Steven)*

*"I never have much time because I’m running around after the children" (Lucy)*

*"When you’re caring for someone your routine is around the person you are caring for" (Charles)*

*"It’s the time as well, because you are sort of restricted, I’m in the office 9-5 there is only so much I can do. I can go out at lunchtime and maybe more activities in the evening but it is just fitting it all in"* (Lucy)

*"It kept buzzing at me to do more steps and it was raising awareness of how lazy… my job doesn’t help me though because I’m sat in an office dealing with people. People that are office based that will really indicate to them how little you move about"* (Evie)

*"If I’m presenting I can’t be looking at my watch, if I’m in a meeting it just, sorry I can’t be looking at my phone and if I’m in a meeting I can’t be looking at my phone too much and in social settings people have said it’s rude to keep looking at your phone, put it down"* (Arthur)

*"I did feel tired but I did feel energised, even though I went out so now I just think I’ve been sat in the office, I should go out and then you see the weather and think no, forget it. I’m not going out in the rain… I didn’t lose the interest, I was just like, do I have to get up and go now. It was more like the weather, I was thinking I know I have to do a few more steps but I can’t be bothered because it’s raining"* (Lucy)

*"Aware of how inactive I was, yes. In the winter, I am almost a hermit because I can’t go out with the cold. This weather is fine but once it gets too hot then its dry and my throat dries up. I don’t really get any real benefit after that. I did notice when I was in Australia, I’ve been there several times with the kids that I can go anywhere, although it’s hot, it’s also a little bit more moist than we get here when it’s hot. I can walk wherever I want, provided I can do it at my own pace"* (Noah)

*"If you’re sitting with somebody in front of you, you can’t suddenly say, ‘I’ve just got to go and walk 250 steps’"* (Joseph)
